# Lessons learnt from the clinico-genomic profiling of families with Li Fraumeni syndrome at a tertiary care centre in North India

**DOI:** 10.3332/ecancer.2023.1550

**Published:** 2023-05-11

**Authors:** Ghazal Tansir, Sameer Rastogi, Sravan Kumar Dubasi, Sindhu Chitikela, Lavu Rohit Reddy, Adarsh Barwad, Ankur Goyal

**Affiliations:** 1 Department of Medical Oncology, All India Institute of Medical Sciences, New Delhi 110029, India; 2 Department of Medical Oncology, Yashoda Hospitals, Hyderabad, Telangana 500024, India; 3 Department of Pathology, All India Institute of Medical Sciences, New Delhi 110029, India; 4 Department of Radiodiagnosis, All India Institute of Medical Sciences, New Delhi 110029, India

**Keywords:** genetic syndromes, genetic counselling, hereditary cancers, sarcoma

## Abstract

Li Fraumeni syndrome (LFS) is an inherited cancer predisposition syndrome due to TP53 gene mutation. There is sparse literature on LFS in the Indian population. We conducted a retrospective study of patients diagnosed with LFS and their family members, registered at our Medical Oncology Department between September 2015 and 2022.

9 LFS families consisted of 29 patients diagnosed currently or historically with malignancies including 9 index cases and 20 first or second-degree relatives. Of these 29 patients, 7 (24.1%) patients developed their first malignancy before the age of 18 years, 15 (51.7%) were diagnosed between 18and and 60 years, and 7 (24.1%) were diagnosed at age more than 60 years. A total of 31 cancers occurred among the families, including 2 index cases who had metachronous malignancies. Each family had a median of three cancers (range 2–5); sarcoma (*n* = 12, 38.7% of total cancers) and breast cancer (*n* = 6, 19.3% of total cancers) being the commonest malignancies. Germline TP53 mutations were documented among 11 patients with cancers and 6 asymptomatic carriers. Of these nine mutations, the most common types were missense (*n* = 6, 66.6%) and nonsense (*n* = 2, 22.2%), and the commonest aberration was replacement of arginine with histidine (*n* = 4, 44.4%). Eight (88.8%) families met either classical or Chompret’s diagnostic criteria and two (22.2%) satisfied both. Two (22.2%) families fit the diagnostic criteria prior to onset of malignancy in the index cases but were untested till the index cases presented to us. Four mutation carriers from three families are undergoing screening as per the Toronto protocol. No new malignancies have been detected so far during the mean surveillance duration of 14 months.

The diagnosis of LFS has socio-economic implications for patients and their families. Delay in genetic testing misses out a crucial window wherein asymptomatic carriers could initiate surveillance in a timely fashion. Greater awareness on LFS and genetic testing in Indian patients is warranted for better management of this hereditary condition.

## Background

Li Fraumeni syndrome (LFS) is an inherited cancer predisposition syndrome which results from the pathogenic germline mutation of the TP53 gene [1]. The p53 protein functions as a tumour suppressor and leads to upregulation of cell cycle arrest, deoxyribonucleic acid (DNA) damage repair and apoptosis [2]. In the presence of deleterious mutations of the TP53 gene, recurrent sublethal and mutagenic DNA alterations accumulate and lead to a heightened susceptibility to various types of malignancies. LFS encompasses a wide spectrum of cancers, including breast cancers, soft tissue sarcomas (STS), brain tumours, leukaemias, adrenocortical carcinomas (ACC) [3]. This heightened predisposition to develop malignancies leads to a cumulative cancer risk of 50% by 46 years which escalates to nearly 100% by the age of 70 years [4].

The entity of LFS came into recognition following the decisive contribution by Frederick Li and Joseph Fraumeni following their study on 641 children with rhabdomyosarcomas [5]. This led to the recognition of four families with a significant family history of multiple malignancies [6], which were followed up for a period of 12 years. This culminated into the term ‘Li Fraumeni syndrome’ first used in 1982 [7] and the description of the classical definition of the syndrome in 1988 [3]. Other clinical criteria which are less stringent than the classical definitions include the Birch and Eeles’ criteria to encompass an LFS-like phenotype [8, 9]. A 2009 modification by the French LFS working group has led to the formulation of the Chompret criteria which are more inclusive of the different clinical presentations associated with the syndrome [10]. The Chompret criteria have a sensitivity and specificity of 82%%–95% and 47%%–58%, respectively, and include any one of the following clinical scenarios [11]:

1. Index case with a tumour belonging to the LFS spectrum (STS, breast cancer, osteosarcoma, brain tumours, leukaemias, ACC, bronchoalveolar lung cancer) with one first- or second-degree relative with an LFS-related tumour except breast cancer if the individual has breast cancer before the age of 56 years.
2. Multiple primary tumours in an individual (except multiple breast tumours) with onset of the first cancer before the age of 46 years, two of which belong to the LFS spectrum.
3. Rare cancers such as ACC or choroid plexus carcinoma, irrespective of family history.

The incidence of LFS among the Western population has been described to be 1:2,000–5,000, with the Brazilians harbouring the founder TP53 germline mutation, p.R337H variant at a frequency of 1:300 [12]. The increasing use of multigene panel-based assays as part of evaluation of malignancies is leading to greater recognition of the germline TP53 mutations even in those families that do not fulfil the LFS diagnostic criteria. However, data on LFS among the Indian population is scarce and only in the form of few published case reports. This may stem from limited testing resources, inadequate awareness among medical practitioners about when to suspect LFS and resistance to genetic testing among patients and their families. It is known that patients and families affected with LFS experience psychosocial issues with multiple studies reporting feelings of anxiety and grief among them [13]. Treating haematologists, oncologists and genetic professionals must be prepared to help patients manage the complex clinical and psychosocial aspects of this condition.

We have conducted this retrospective study on the clinical, epidemiological and genetic profile of the patients and their families diagnosed with LFS who presented to our oncology clinic. This study is the only case series from India so far, to describe families with LFS and offers a look into the unique problems faced during their counselling and management.

## Methods

We retrospectively evaluated the records of patients with LFS who were registered at our sarcoma medical oncology clinic between September 2015 and 2022. The patients included were diagnosed with LFS using the Chompret’s and/or classical criteria, at either our centre or elsewhere. Details of the patients’ clinical and epidemiologic profile, family history, treatment received and outcomes were noted from the hospital files. The family history was established in the form of a three-generation pedigree with the details of deceased cancer patients in the family done by verification of prior hospital records. In the case the historical medical documents detailing the tumour profile were not available, the tumour site and type were noted as per the history provided. The deceased cancer patients for whom the type and/or site of disease were not available were classified under ‘unknown aetiology’.

The testing for germline TP53 mutation for the patients and family members had been performed at College of American Pathologists accredited laboratory (MedGenome laboratory) by next-generation sequencing (NGS). The mutations were reported as pathogenic/likely pathogenic (P/LP) using the ClinVar database [14]. Information on the TP53 genetic mutations obtained included the type of mutation, the exons and codons involved and the alteration in amino acids. These data were sought after clearance from the Institutional Ethics Committee (IEC-449/02.07.2021). The statistical analysis was performed via SPSS 26.0 (SPSS, Chicago, IL, USA). Nominal data is provided as number (%) and the continuous data as median and mean values as applicable.

## Results

**Figure S1.**
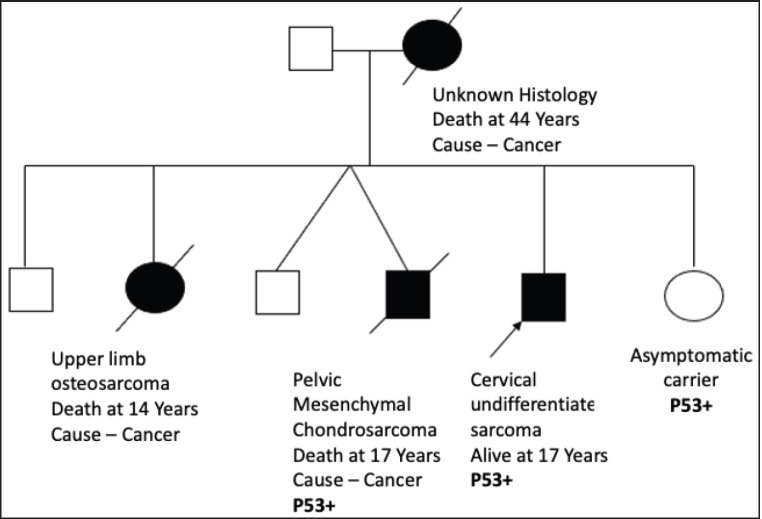
Pedigree chart of Family 1 (Germline TP53 mutation: c.1024C>T [p.Arg342Ter]).

**Figure S2.**
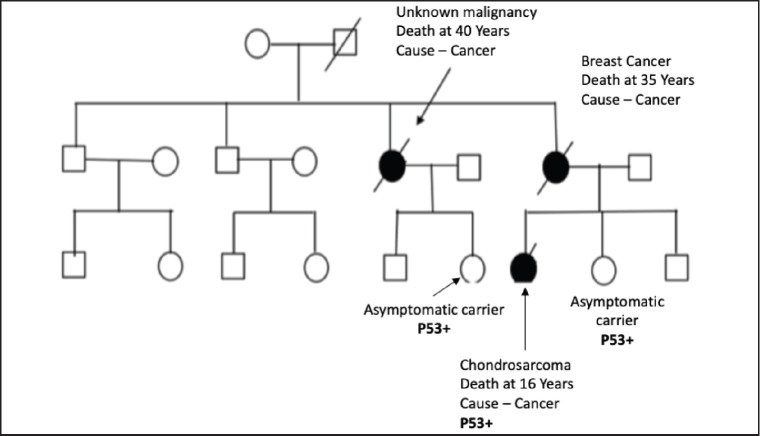
Pedigree chart of Family 2 (Germline TP53 mutation: c.524G>A [p.Arg175His]).

**Figure S3.**
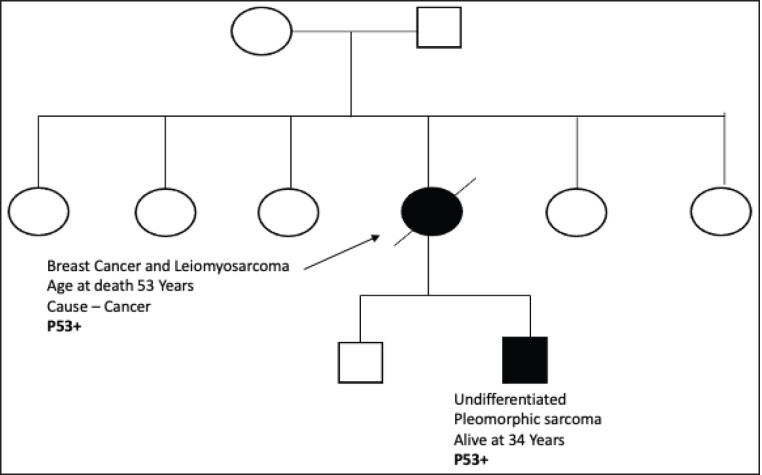
Pedigree chart of Family 3 (Germline TP53 mutation: c.520C>T [p.Arg174*]).

**Figure S4.**
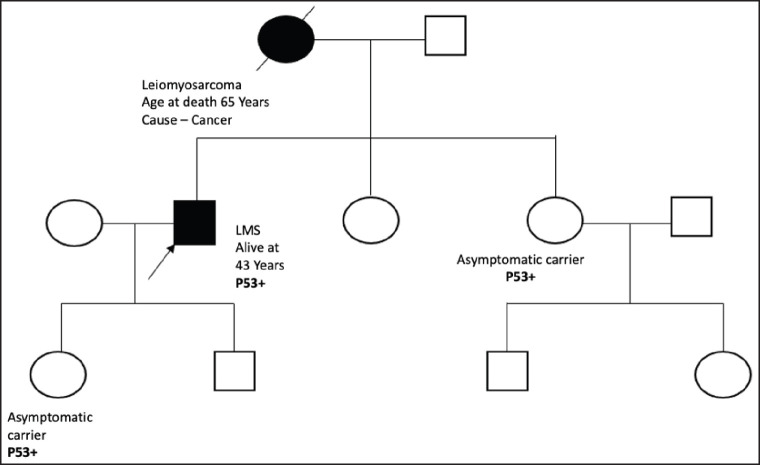
Pedigree chart of Family 4 (Germline TP53 mutation: c.473G>A [p.Arg158His]).

**Figure S5.**
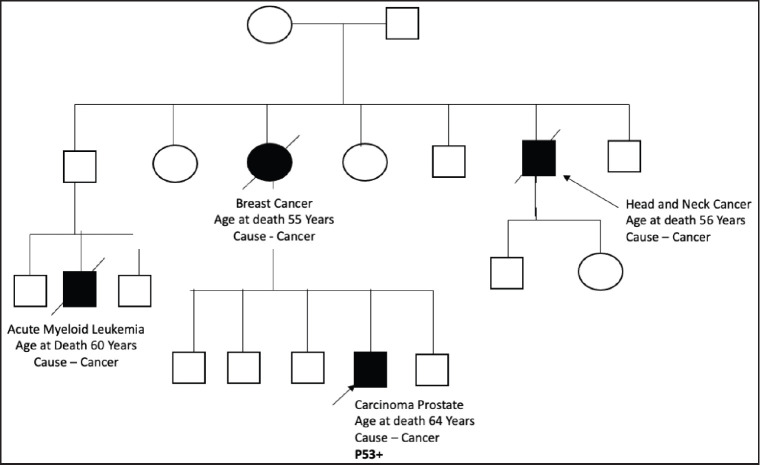
Pedigree chart of Family 5 (Germline TP53 mutation: c.818G>A [p.Arg273His]).

**Figure S6.**
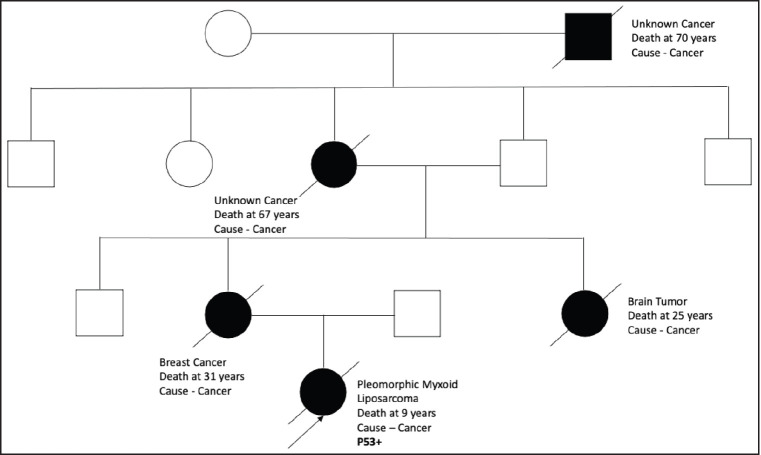
Pedigree chart of Family 6 (Germline TP 53 mutation: c.636del [p.Arg213AspfsTer34]).

**Figure S7.**
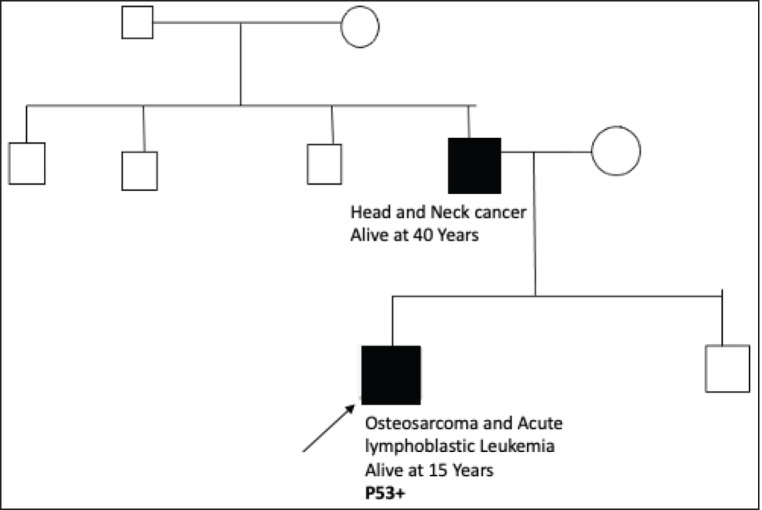
Pedigree chart of Family 7 (Germline TP53 mutation: c.743G>A [p.Arg248Gln]).

**Figure S8.**
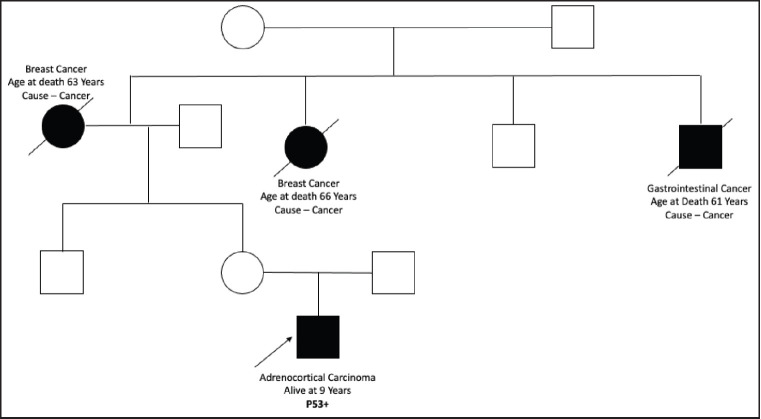
Pedigree chart of Family 8 (Germline TP53 mutation: c.641A>G [p.His214Arg]).

**Figure S9.**
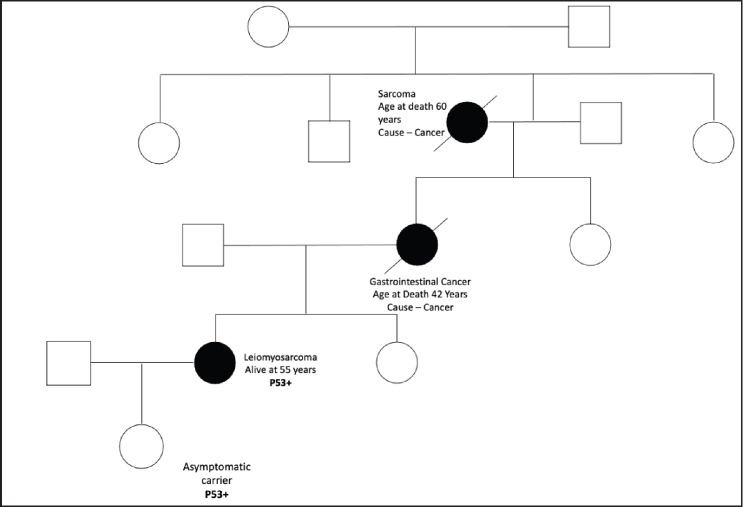
Pedigree chart of Family 9 (Germline TP53 mutation: c.473G>A [p.Arg158His]).

**Figure S10.**
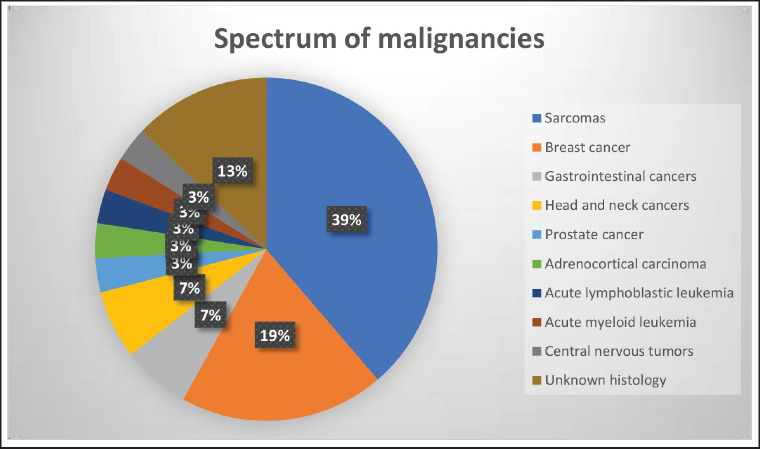
Distribution of malignancies in the subjects included in the study population in families diagnosed with lfsLFS.

We identified 9 families having LFS consisting of 29 subjects diagnosed with malignancies, including 9 index cases and 20 first or second-degree relatives (Figures S1–S9). There were a total of 31 current or historic diagnoses of malignancies which included 11 cancers in the index cases and 20 cancers in the family members (Table 1). Two index cases had experienced more than one malignancy, and the overall cohort had a median of three cancers (range 2–5) per family. The overall cohort comprised 13 (44.8%) males and 16 (55.2%) females with the median age at onset of malignancy being 43 years (range 9–70). The index cases included six (66.6%) males and three (33.4%) females and the median age at onset of malignancy among them was 17 years (range 9–63). The age distribution of onset of first malignancy was less than or equal to 18 years in 7 (24.1%) patients, between 18 and 60 years in 15 (51.7%) patients, and greater than 60 years in 7 (24.1%) patients. The most common cancers that were diagnosed in the families were sarcomas (*n* = 12, 38.7% of total 31 cancers), breast cancer (*n* = 6, 19.3%), gastrointestinal cancers and head neck squamous cell cancer (*n* = 2, 6.4% each). The spectrum of malignancies is detailed in Table 2 and Figure S10.

29 individuals comprising of patients alive with malignancies and their asymptomatic family members underwent genetic testing for hereditary germline mutations. Among them, 11 patients with cancers (including 9 index cases) and 6 asymptomatic relatives were found to be positive for germline TP53 mutation-positive. All the TP53 mutations were classifiable as P/LP according to the ClinVar mutation database (Table 3). These included point mutations (*n* = 8, 88.9%) and frameshift mutation (*n* = 1, 11.1%). The subtypes of point mutations included missense mutations (*n* = 6, 66.67%) and nonsense mutations (*n* = 2, 22.2%). The distribution of involved exons ranged from 5 to 11, most commonly at exon 5 (*n* = 3, 33.3%).

Eight (88.8%) families met either of the classical or Chompret criteria for LFS, while two (22.2%) met both. Three (33.3%) families met the individual classical criteria and seven (77.7%) met the Chompret’s criteria. Five index cases (55.5%) had met the diagnostic criteria at the time of initial management of malignancy at other centres. But they underwent genetic testing after a delay of median 8 months (range 3–30) upon presentation to our hospital. Two families (22.2%) satisfied the diagnostic criteria even prior to development of the malignancy in the index cases, but remained untested until the index cases acquired cancers that were managed at our department. One family had initially denied genetic testing despite having met the diagnostic criteria, and only agreed to it when a kin developed malignancy.

Currently, four asymptomatic carriers of three (33.3%) families are undergoing screening as per the Toronto protocol. Of these, one family had reported financial difficulty during surveillance as insurance cover is not available for cancer screening procedures. For the other six families, the reason(s) for not following the screening regimen included refusal for genetic testing by the remaining family members (*n* = 4), no surviving first or second-degree members (*n* = 1), and dropping out after 1 year of screening due to social ostracisation (*n* = 1). After a mean duration of 14 months of surveillance, no malignancies have been detected in the four asymptomatic mutation carriers. The index cases currently alive (*n* = 5, 55.5%) are undergoing routine imaging as part of management of their primary malignancies, and no new cancers have developed in them so far.

## Discussion

This is the first Indian case series to portray the spectrum of clinical presentations and TP53 mutations observed among the patients diagnosed with LFS. The striking tendency to develop cancers in LFS is reflected from the large proportion of patients who develop their first cancers at young ages. More than one-fifth of our patients developed their first malignancies by the age of 18 and 75.8% did so by the age of 60 years, which is in agreement by the observations by Mai *et al* [4]. The median age of tumour onset in the index cases of 17 years is lesser than that described previously [15] Multiple cancers and therapy-related malignancies have been reported in previous studies, with Mai *et al* [4] describing 49% of germline TP53 mutation carriers who had second malignancies after a median period of 10 years. Two patients in our study population have developed second malignancies, including leiomyosarcoma and acute lymphoblastic leukaemia (ALL) in one patient each.

Among the tumour subtypes, we found a predominance of sarcomas (35.4%) rather than breast cancer (19.3%). This finding contrasts with those described by Bougeard *et al* [10] wherein 60% of total 332 patients had breast cancers while STS and osteosarcoma were the next two common diagnoses. This difference could be attributed to our patient population which included those cases who had presented first to the sarcoma clinic of our department. The other common tumours, namely head and neck cancer and gastrointestinal cancers (6.4% each of total cancers in our study) were also noted in 2% and 5% of patients each in previous studies [16]. While ACC and brain tumours have been described to constitute 6%–13% and 9%–14% of the cancer diagnoses [17], they were found only in one patient each (3.2% of total cancers) among our cohort. Leukaemias, including ALL and acute myeloid leukaemia (AML), have been found in 2%–4% of germline TP53 mutation carriers [10]. ALL in LFS patients classically have a pre-B cell hypodiploid phenotype [18] and conversely, 19% of children with hypodiploid ALL have germline TP53 mutations [19]. However, the single case of paediatric leukaemia (ALL) in our series had a pro-B ALL phenotype with a normal karyotype.

A study of the International Agency for Research on Cancer (IARC) database found that 70.5% of the 3034 TP53 mutation-positive patients had fulfilled the LFS genetic testing criteria or had developed a malignancy prior to the age of 18 years [20]. The fulfilment of either classical or Chompret’s testing criteria in 88% of our study population is also higher in comparison with these findings. While 82.2% of LFS mutations were P/LP in the IARC database, all mutations were found P/LP among our patients. These findings can be explained by the fact that our study population is limited in number and restricted to diagnosed cancer patients only.

The mutational spectrum consisted of point mutations in approximately 90% of patients, which is higher than the 67% reported by Bougeard *et al* [10]. However, the exon distribution of the point mutations from exons 5 to 11 matches the findings of this particular study. Although we could not identify multiple codon ‘hotspots’ as observed by Wasserman *et al* [21], codon 158 was found involved in 2 (22.2%) of our families. Owing to the limited number of patients in this study, we could not ascertain any genotype-phenotype correlation. However, this phenomenon has been recognised previously among larger databases. Dominant-negative missense mutations have been associated with earlier tumour onset while the less severe alterations such as nonsense mutations, frameshift mutations or genomic arrangements present with tumours at a later age [10]. Also, the Brazilian founder mutation of TP53 R337H accords a lower penetrance and causes mainly childhood ACC [22].

Regular cancer surveillance as per the Toronto protocol has been found to accord a significant overall survival (OS) benefit in LFS with a 5-year OS of 88.8% compared with 59.6% in non-screened patients [23]. But studies have found a high incidence of clinically relevant distress and anxiety while undergoing cancer screening in patients affected with LFS [24]. The cost-effectiveness of cancer surveillance in LFS has been proven cost-effective in the real-world but the limited insurance cover available currently poses financial hurdles for the affected families [12, 25]. Due to limited resources and lesser awareness among medical practitioners and patients, these challenges are greater in the developing world. This is reflected in our study by the delayed diagnosis of LFS among five families where the index cases were being managed at non-expert centres initially. This missed opportunity also comes to fore when two families were already fitting the diagnostic criteria but were only investigated when a new cancer developed and the patients presented to us for the same. The refusal for testing by other family members despite knowing the hereditary nature of this disease, denial for screening procedures and dropouts due to social stigmas and exclusion were other challenges against regular cancer surveillance.

The limitations of our study include the constraints of its retrospective design and small sample size. We acknowledge the selection bias for our cohort because our study was based at the sarcoma division of our department. Due to this, there was predominance of sarcomas as opposed to breast cancers, which are otherwise the more prevalent malignancy. It is also imperative to note that certain malignancies such as carcinoma prostate in the index case of Family 5 could be incidental rather than associated with the germline TP53 mutation. With the increasing use of NGS and mutational profiling, germline mutations could also be incidentally diagnosed during evaluation of probably non-hereditary cancers. Our profile of Indian LFS patients gives significant directions for the conduct of future studies in this domain. More Asian data need to be generated regarding the profile of TP53 mutations in patients and their family members, genotype–phenotype correlations with different types of mutations, and outcomes with surveillance measures. Prospective psychosocial assessment and appropriate early interventions will be required for mitigation of the impact that the diagnosis of this hereditary cancer syndrome has on a family.

## Conclusion

This study presents the largest set of Indian clinico-genomic data on patients with LFS and the challenges faced during their management and follow-up. We could identify the common malignancies, the types of TP53 mutations prevalent in these patients and highlight the need for larger prospective and multicentre trials in this domain.

## List of abbreviations

DNA: Deoxyribonucleic acid.

## Conflicts of interest and funding

The authors declare that they have no conflict of interest. The authors certify that they have no affiliations with or involvement in any organisation or entity with any financial interest such as honoraria; educational grants; participation in speakers’ bureaus; membership, employment, consultancies, stock ownership or other equity.

## Author contributions

Study concept/design: GT/SR/SKD/LRR

Data acquisition: AB/AG

Data analysis and interpretation: GT/SR

Manuscript preparation: GT/SR

Manuscript editing/reviewing: GT/SR

## Disclosure of results at a meeting

Results from a smaller dataset of this population were shared in the form of abstract and poster at the European Society of Medical Oncology (ESMO) Asia 2022 meet held at Singapore in December, 2022 [26].

## Ethical approval

The study protocol was approved by the Institute Ethics Committee vide letter number IEC-449/02.07.2021.

## Availability of data material

Data regarding this study will be available from the corresponding author on reasonable request.

## Figures and Tables

**Table 1.** Clinical details and spectrum of malignancies in the study population.

Family	Malignancy	Gender (male/female)	Age at first malignancy (years)	Satisfied classical criteria (yes/no)	Satisfied Chompret’s criteria (yes/no)	Mutation as per ClinVar classification^14^ (type of mutation)
Family 1	Undifferentiated sarcoma* (index case)	Male	17	Yes	Yes	c.1024C>T [p.Arg342Ter] (Nonsense)
	Chondrosarcoma* (brother)	Male	16			
	Osteosarcoma^ϕ^ (sister)	Female	15			
	Unknown histology^ϕ^ (mother)	Female	43			
Family 2	Chondrosarcoma* (index case)	Female	16	Yes	No	c.524G>A [p.Arg175His] (Missense)
	Breast cancer^ϕ^ (mother)	Female	35			
	Unknown^ϕ^ (maternal aunt)	Female	40			
Family 3	Breast cancer, leiomyosarcoma* (index case)	Female	50	No	Yes	c.520C>T [p.Arg174*] (Nonsense)
	Undifferentiated pleomorphic sarcoma* (son)	Male	30			
Family 4	Leiomyosarcoma* (index case)	Male	43	No	Yes	c.473G>A [p.Arg158His] (Missense)
	Leiomyosarcoma^ϕ^ (mother)	Female	65			
Family 5	Carcinoma prostate* (index case)	Male	63	No	No	c.818G>A [p.Arg273His] (Missense)
	AML^ϕ^ (cousin)	Male	60			
	Breast cancer^ϕ^ (mother)	Female	55			
	Head and neck cancer^ϕ^ (uncle)	Male	56			
Family 6	Pleomorphic myxoid liposarcoma* (index case)	Female	9	Yes	Yes	c.636del [p.Arg213AspfsTer34] (frameshift)
	Breast cancer^ϕ^ (mother)	Female	31			
	Brain tumour ^ϕ^ (aunt)	Female	25			
	Unknown malignancy^ϕ^ (grandmother)	Female	67			
	Unknown malignancy^ϕ^ (great grandfather)	Male	70			
Family 7	Osteosarcoma, ALL* (index case)	Male	15	No	Yes	c.743G>A [p.Arg248Gln] (Missense)
	Head and neck cancer (father)	Male	40			
Family 8	ACC* (index case)	Male	9	No	Yes	c.641A>G [p.His214Arg] (Missense)
	Breast cancer^ϕ^ (maternal grandmother)	Female	63			
	Breast cancer^ϕ^ (sister of maternal grandmother)	Female	66			
	Gastrointestinal cancer^ϕ^ (brother of maternal grandmother)	Male	61			
Family 9	Leiomyosarcoma* (index case)	Female	55	No	Yes	c.473G>A [p.Arg158His] (Missense)
	Gastrointestinal cancer^ϕ^ (mother)	Female	42			
	Sarcoma^ϕ^ (grandmother)	Female	60			

* Patients positive for germline TP53 mutation

ϕ Patients deceased and untested at the time of analysis

**Table 2.** Details of age at diagnosis of first malignancy and spectrum of cancers in the study population.

Parameter	Details (*n*, %)
Age at diagnosis of first malignancy	Less than 18 years (7, 24.1%) 18–60 years (15, 51.7%) More than 60 years (7, 24.1%)
Subtypes of malignancies	Sarcomas (12, 38.7%) Breast cancer (6, 19.3%) Gastrointestinal cancers (2, 6.4%) Head and neck cancers (2, 6.4%) Prostate cancer (1, 3.2%) ACC (1, 3.2%) ALL (1, 3.2%) AML (1, 3.2%) Central nervous system tumour (1, 3.2%) Unknown histology (4, 12.8%)

**Table 3.** Details of TP53 mutations in the study population.

Parameter	Detail (*n*, %)	
Type of mutation	Missense (6, 66.7%) Nonsense (2, 22.2%) Frameshift mutation (1, 11.1%)	
Exon	5 (3, 33.3%) 6 (1, 11.1%) 7 (1, 11.1%) 8 (1, 11.1%) 10 (1, 11.1%) 11 (1, 11.1%) Not known (1, 11.1%)	
Codon and corresponding variant amino acid	158 (2, 22.2%) 174 (1, 11.1%) 175 (1, 11.1%) 213 (1, 11.1%) 214 (1, 11.1%) 248 (1, 11.1%) 273 (1, 11.1%) 342 (1, 11.1%)	Arginine to histidine Arginine to termination Arginine to histidine Arginine to termination Histidine to arginine Arginine to glutamine Arginine to histidine Arginine to termination
